# Taste alterations in breast cancer patients undergoing chemotherapy: an observational study

**DOI:** 10.3389/fnut.2026.1815891

**Published:** 2026-04-22

**Authors:** Andrea Devecchi, Valentina Casalone, Valentina Ponzo, Dora Tortarolo, Anna Sapino, Simona Bo

**Affiliations:** 1 University of Gastronomic Sciences of Pollenzo, Pollenzo, Italy; 2 Department of Medical Sciences, University of Turin, Turin, Italy; 3 Clinical Nutrition and Dietetics Unit, Candiolo Cancer Institute, FPO-IRCCS, Candiolo, Italy; 4 Department of Computer Science, University of Turin, Turin, Italy; 5 Pathology Unit, Candiolo Cancer Institute, FPO-IRCCS, Candiolo, Italy

**Keywords:** breast cancer, chemotherapy, dysgeusia, objective methods, subjective methods, taste, taste alterations

## Abstract

**Background:**

Taste alterations are a common adverse effect of chemotherapy and may substantially impair quality of life, social interactions, and dietary habits. Reported incidence rates vary widely, partly due to the heterogeneity of assessment methods. This study aimed to investigate chemotherapy-induced taste alterations in patients with breast cancer using both objective (taste strips) and subjective (questionnaire-based) measures.

**Materials and methods:**

This was a prospective study conducted between July 2023 and June 2025 at the Candiolo Cancer Institute FPO-IRCCS, in Torino (Italy). Sociodemographic, clinical, anthropometric variables, and dietary habits were collected. Taste alterations were evaluated using both subjective and objective assessment tools (taste strips).

**Results:**

A total of 94 female patients were enrolled. Of these, 70 patients had completed chemotherapy and taste evaluation and were included in the statistical analyses. According to subjective assessment, all taste modalities were significantly altered from pre- to post-chemotherapy evaluation, whereas one third of patients exhibited taste impairment when assessed using taste strips. The two methods showed limited agreement. In multiple regression models adjusted for age, smoking status, and pack-years, higher education level was inversely associated with subjective deterioration in sour (*β* = −0.49; 95% CI −0.82, −0.16; *p* = 0.006) and bitter taste perception (*β* = −0.47; 95% CI −0.79, −0.13; *p* = 0.006). Moreover, carbohydrate intake was inversely associated with worsening of overall taste perception (*β* = −1.96; 95% CI −3.72, −0.20; *p* = 0.035), as well as sour (*β* = −2.05; 95% CI −4.01, −0.09; *p* = 0.044) and salty taste perception (*β* = −1.92; 95% CI −3.76, −0.04; *p* = 0.046), based on subjective assessment.

**Conclusion:**

Taste alterations affect a substantial proportion of patients with breast cancer undergoing chemotherapy. The low agreement observed between subjective and objective measures suggests that these methods may assess distinct aspects of taste dysfunction and should be interpreted accordingly.

## 1 Introduction

Taste alterations (TAs) are a common and distressing side effect of chemotherapy, affecting approximately 50–80% of breast cancer (BC) patients, particularly those treated with taxanes and anthracyclines (1–3). TAs can manifest as hypogeusia (partial loss of taste), ageusia (complete loss of taste), or dysgeusia, defined as a qualitative distortion of taste perception in the presence of a stimulus (4). Alterations in different taste modalities (sweet, salty, bitter, sour, and umami) have been reported; however, their extent and pattern vary across studies, and not all taste qualities are consistently affected. Among these, salty taste is often described as the most impaired (2, 3, 5).

Taste alterations are frequently associated with changes in dietary habits in BC patients (6, 7), with dysgeusia and food availability representing key determinants of nutritional choices during chemotherapy (7). However, food perception in cancer patients is a multisensory process. In addition to taste and smell, alterations in chemesthetic, thermal, and somatosensory perception, as well as impaired salivary function, may influence eating behaviour (8, 9). In particular, somatosensory alterations have been identified as important determinants of food perception alongside gustatory and olfactory dysfunction (10).

These sensory changes may contribute to heterogeneous nutritional outcomes. Some patients experience weight gain and a more pro-inflammatory dietary profile (11), while others develop reduced food intake and weight loss, with approximately 20% of BC patients being undernourished or experiencing >10% weight loss during the disease course (12). Moreover, TAs have been associated with reduced appetite, loss of the hedonic aspects of eating, and impaired quality of life (5, 12).

Taste alterations can be assessed using subjective and objective methods. Subjective tools include questionnaires such as the Chemotherapy-Induced Taste Alteration Scale (CiTAS) and symptom-based scales (13, 14, 41). Objective methods include electro-gustometry, liquid taste solutions, filter paper strips, and food-based models, which may capture different aspects of sensory perception and eating experience (15–17).

Most studies have relied on subjective assessments, whereas only a limited number have combined subjective and objective approaches in BC patients, often reporting discrepancies between the two methods (1, 18). A recent study further highlighted the complementary nature of these approaches, showing that objective measures provide insight into sensory function, while subjective assessments better reflect patients’ experiences and food-related behaviours (19).

The present study aimed to investigate the incidence and characteristics of taste alterations in BC patients undergoing chemotherapy by combining subjective (validated questionnaire) and objective (taste strips) methods and evaluating the agreement between them.

## 2 Materials and methods

### 2.1 Participants

This was a prospective study conducted between July 2023 and June 2025 at the Candiolo Cancer Institute FPO-IRCCS, in Torino (Italy). Inclusion criteria included diagnosis of breast cancer, age between >18 and <70 years; ability to provide written informed consent to participate in the study; indication for neoadjuvant and adjuvant chemotherapy. Exclusion criteria were inability to guarantee participation in follow-up visits; allergy to the components of the taste strips; pregnancy; pre-existing dysgeusia; indication for palliative chemotherapy; metastatic disease; previous treatment for head and neck cancers; previous treatment with chemotherapy.

### 2.2 Ethical considerations

The study protocol was approved by the Ethics Committee of Candiolo Cancer Institute FPO-IRCCS in April 2023 (protocol 70/2023). Informed consent was obtained from all participants before data collection. Questionnaires addressing socio-demographic and anthropometric characteristics, as well as those concerning taste alterations and dietary habits, were developed using the Qualtrics® platform (Qualtrics, Provo, UT), which adheres to internal standards of privacy and data protection.

### 2.3 Measurements

The following data were collected at enrolment (T0) before chemotherapy starting and after its completion (T1):

- Age, education level, smoking habits, presence of allergies, duration of chemotherapy, type of chemotherapy, any concomitant therapies, comorbidities, surgical interventions for BC;
- Questionnaire for the assessment of TAs (Chemotherapy-Induced Taste Alteration Scale - CiTAS) (20);
- Semi-quantitative food-frequency (FFQ) questionnaire (21);
- Measurements of weight, height, and arm circumference.

Tas were assessed by taste strips immediately after the completion of chemotherapy. The timing of assessments was selected to capture the cumulative and peak effects of chemotherapy-related toxicity on taste perception and is consistent with previous longitudinal studies (2, 3).

The test was conducted at hospital in the morning under fasting conditions by a trained healthcare professional (VC, AD) following the manufacturer’s instructions.

Initially, objective taste assessments were planned at both baseline (T0) and post-chemotherapy (T1). However, during the initial phase of the study, a potential familiarization effect with the taste strips was observed, with improved performance at repeated testing that could not be clearly distinguished from true changes in gustatory function. To minimize this potential bias and preserve the internal validity of the post-treatment assessment, objective taste testing was subsequently performed only at T1.

### 2.4 Questionnaires

A form containing all the above-described questionnaires and data was created by the Qualtrics platform and was administered by trained healthcare professionals (VC, AD).

The validated Italian version of the CiTAS questionnaire (20) was employed. It consists of 18 items grouping into:

a) Quantitative changes in taste perception (hypogeusia and ageusia)
b) Qualitative changes (heterogeusia and cacogeusia)
c) Problems related to the sphere of nutrition such as difficulty eating hot or high-fat foods.

The items are evaluated using a five-point Likert scale, where 1 corresponds to “no difficulty or absence of the disorder” and 5 to “maximum difficulty or very much disturbance.” The CiTAS questionnaire was administered and scored according to its original validated structure. Only the ‘Taste Diminution’ subscale of the CiTAS was analyzed, as it represents the only subjective dimension directly comparable to the sensory thresholds assessed via Taste Strips. No additional factor analysis was performed in the present sample, and the original item structure was retained to ensure comparability with previous studies.

A validated semi-quantitative FFQ consisting of 36 items and assessing portion sizes and weekly consumption frequencies was used (21). Food items were grouped into categories including beverages, dairy products, meat, fish, eggs, cereals, vegetables, legumes, fruits, fatty condiments, and other foods (e.g., sweets, fried foods, fast food). Alcohol, soft drinks, and coffee intake were also recorded (21). Food photographs as illustrative examples to facilitate the estimation of portion sizes were employed. Individual daily nutrient intakes were estimated based on the average nutrient composition (expressed as kcal, proteins, lipids, carbohydrates, sugars, alcohol, and dietary fiber) of each food group according to Italian food composition databases (22).

### 2.5 Anthropometric measurements

Body weight and height were measured using a professional scale equipped with an integrated stadiometer (GIMA Pegaso Eletronic Body Scale). Mid-upper arm circumference was measured using a flexible, non-elastic tape on the non-dominant arm. The midpoint between the acromion and the olecranon was identified with the arm relaxed alongside the body. The tape was placed horizontally around the arm at this point, ensuring it was snug but not compressing the skin.

### 2.6 Taste strips

The taste strips are 8 cm-length strips of paper impregnated with substances relative to sweet, salty, sour, and bitter taste (Burghart, Wedel, Germany). For each taste modality, four concentrations of increasing intensity were employed: sweet (sucrose: 0.05, 0.1, 0.2, 0.4 g/mL), sour (citric acid: 0.05, 0.09, 0.165, 0.3 g/mL), salty (sodium chloride: 0.016, 0.04, 0.1, 0.25 g/mL), and bitter (quinine hydrochloride: 0.0004, 0.0009, 0.0024, 0.006 g/mL). The taste strip test was administered under participant blinding using a manufacturer-provided randomization scheme (Supplementary Figure S1). Patients refrained from eating, smoking, or using chewing gum for at least one hour before, with water allowed. Strips were sequentially placed on the tongue using specialized tweezers, and participants moved the tongue to mix the substance with saliva before reporting the perceived taste. Mouth rinsing with water was performed between strips. Control strips without taste were included. The maximum score corresponds to 16, i.e., one point for each taste identified, as there were 4 concentrations for four tastes. Neutral stripes did not provide a score. A score ≥9 indicated normogeusia, while a score <9 indicated hypogeusia. For individual taste modalities, failure to identify any concentration was classified as ageusia for that specific taste. Identification of a single concentration was considered a case of false recognition, except for the bitter taste, for which correctly identifying even one of the four concentrations was considered accurate taste perception. Normative values for the Taste Strips test are derived from the studies by Mueller et al. (23) and Landis et al. (24). These thresholds are widely used in clinical research to classify taste impairment, although they were originally established in non-oncological populations.

It is essential to emphasize that the CITAs questionnaire score and the taste strip score follow opposite trends. In the questionnaire, lower scores correspond to better taste perception, whereas in taste strip assessments higher scores indicate better perception.

### 2.7 Statistical analysis

Results were expressed as mean and standard deviation for quantitative variables and as frequency distribution for categorical variables. Changes in scores of CITAs questionnaire and in food intakes were expressed as delta values (values at T1 – values at T0).

Variable distribution was evaluated by the Shapiro–Wilk test. Differences between groups were analyzed using the Mann–Whitney test, the Kruskal–Wallis test, the Wilcoxon signed rank test, the chi-square test, and ANOVA, as appropriate. Spearman’s nonparametric correlation test was used to detect correlations between variables. A multiple linear regression model, adjusted for age, education, BMI, and smoking habit was performed to assess the associations between TAs and clinical variables.

To assess the agreement between subjective and objective methods, the CiTAS and taste strip scores were harmonized to allow direct comparison. Specifically, a rescaled CiTAS score ranging from 0 to 4 was derived to match the range of the objective test (0 = maximal alteration, 4 = best taste recognition). The original CiTAS scores (1 = no alteration, 5 = maximum alteration) were first inverted using the formula: inverted score = (6 − original score), so that higher values indicated better taste perception. The inverted scores were then linearly rescaled to a 0–4 range by subtracting 1. Agreement between the two methods was assessed using Cohen’s weighted kappa with quadratic weights. This linear transformation was applied exclusively for the agreement analysis to ensure congruence in scale direction and range, while preserving the relative ordering of the original CiTAS responses. All other clinical and nutritional analyses involving CiTAS were conducted using the original, validated scoring system to preserve its clinical interpretability.

## 3 Results

### 3.1 Study population

A total of 94 female patients were enrolled. Of these, 24 did not complete the study due to treatment discontinuation, withdrawal, or incomplete assessments. Therefore, 70 patients who completed chemotherapy and taste evaluation were included in the final analysis (Supplementary Figure 2). Participants were middle-aged women with a mean BMI in the overweight range and predominantly omnivorous dietary habits (Table 1).

**Table 1:** Characteristics of participants.

Variables	Mean or %	SD
Age (years)	53.1	9.4
Weight (kg)	67.6	11.3
Height (cm)	161.6	5.9
Body Mass Index (kg/m^2^)	26.0	4.7
Active smoking (%)	21.4	
Place of residence (%)		
City (>70,000 inhabitants)	35.7	
Urban context (10,000–70,000 inhabitants)	32.9	
Rural context (<10,000 inhabitants)	31.4	
Level of education (%)		
Primary schools	20.0	
High schools	40.0	
Bachelor/Master’s degree	40.0	
Dietary habits (%)		
Omnivorous	88.6	
Flexitarian	8.6	
Vegetarian	2.8	
Duration of chemotherapy (months)	4.6	1.2
Comorbidities (%)		
None	50.0	
Autoimmune diseases	5.7	
Cardiovascular diseases	4.3	
Metabolic diseases	2.9	
Psychiatric diseases	4.3	
Multiple comorbidities	27.1	
Others	5.7	

### 3.2 Changes in taste perception and dietary habits

A significant decline in taste perception was observed at the end of chemotherapy compared with baseline, as assessed by the CiTAS questionnaire, both for overall taste perception and for all individual taste modalities (all *p* < 0.001; Table 2). Regarding dietary habits, no significant changes were observed in total energy intake or BMI. However, a modification in dietary composition was detected, with increased protein and fiber intake and reduced sugar and alcohol consumption (Table 2).

**Table 2:** Taste perception scores, clinical and dietary variables at enrolment and at the end of chemotherapy.

Variables	Enrolment (T0)	End of chemotherapy (T1)	*p*-value*
Taste scores (CITAs)			
“Difficulty in tasting food”	1.04 ± 0.20	2.70 ± 1.18	**<0.001**
“Difficulty in perceiving sweet tastes”	1.04 ± 0.20	2.19 ± 1.16	**<0.001**
“Difficulty in perceiving the salty tastes”	1.03 ± 0.17	2.50 ± 1.16	**<0.001**
“Difficulty in perceiving sour tastes”	1.03 ± 0.17	2.10 ± 1.08	**<0.001**
“Difficulty in perceiving bitter tastes”	1.03 ± 0.17	1.96 ± 1.04	**<0.001**
“Difficulty in perceiving umami tastes”	1.03 ± 0.24	2.46 ± 1.22	**<0.001**
Body mass index (kg/m^2^)	26.0 ± 4.7	25.3 ± 6.40	0.360
Dietary intakes			
Energy (kcal/day)	1,368.2 ± 478.4	1,372. ± 399.5	0.282
Protein (% of kcal/day)	16.0 ± 3.0	18.9 ± 3.5	**<0.001**
Lipid (% of kcal/day)	39.2 ± 7.1	38.6 ± 6.4	0.550
Carbohydrates (% of kcal/day)	43.4 ± 8.0	42.1 ± 6.2	0.403
Sugar (% of kcal/day)	12.4 ± 3.9	8.3 ± 2.5	**<0.001**
Alcohol (g/day)	3.5 ± 9.0	0.9 ± 2.2	**0.002**
Fiber (g/day)	19.8 ± 7.8	26.2 ± 10.8	**0.003**

*p-values by Wilcoxon signed-rank test. Statistically significant results are in bold.

### 3.3 Objective assessment of taste alterations

Objective evaluation using taste strips identified hypogeusia in approximately one-third of patients (32.9%). Among taste modalities, sour and salty tastes were the most frequently impaired (30.0 and 17.1%, respectively), while sweet taste was less commonly affected (Table 3). Ageusia was also observed, particularly for salty and sour tastes (21.4 and 17.1%, respectively).

**Table 3:** Results of the assessment of TAs by taste strips at T1.

Outcome	Patients (%)
Overall hypogeusia	32.9
Hypogeusia by taste modalities	
Sweet	7.1
Sour	30.0
Salty	17.1
Bitter	–*
Ageusia by taste modalities	
Sweet	5.7
Sour	17.1
Salty	21.4
Bitter	4.3

*No established normative threshold is available for bitter hypogeusia; therefore, the correct identification of at least one of the four concentrations was considered indicative of preserved taste perception.

### 3.4 Agreement between subjective and objective methods

A moderate agreement between subjective and objective assessments was observed for sweet (*κ* = 0.36, *p* = 0.003) and salty tastes (*κ* = 0.22, *p* = 0.040), whereas no agreement was found for sour taste. Overall agreement across taste modalities was limited (Table 4).

**Table 4:** Weighted Cohen’s kappa (*κ*) values indicating the level of agreement between subjective and objective methods.

Taste	Weighted *κ*	95% CI	*p*-value
Sweet	0.36	0.12; 0.60	**0.003**
Salty	0.22	0.01; 0.43	**0.040**
Sour	−0.003	−0.15; 0.15	0.970
Bitter	0.15	−0.08; 0.39	0.209
General taste	0.21	−0.01; 0.43	0.059

Statistically significant results are in bold.

### 3.5 Associations with clinical and dietary variables

Higher educational level was associated with better taste perception. Specifically, patients with higher education demonstrated better identification of sweet, sour, bitter, and umami tastes by questionnaire (Supplementary Table 1), and better objective perception of salty taste by taste strips [0.5 (IQR: 0–2.75) vs. 2.0 (IQR: 1.0–3.0) vs. 3.0 (IQR: 1.0–3.25), for primary school, high school, and bachelor/master’s degree, respectively; *p* = 0.028]. In multivariable regression models adjusted for age, BMI, and smoking habits, educational level was inversely associated with subjective impairment of sour (*β* = −0.49; 95% CI −0.82 to −0.16; *p* = 0.006) and bitter tastes (*β* = −0.47; 95% CI −0.79 to −0.13; *p* = 0.006), indicating poorer taste perception among patients with lower education.

Smoking was also associated with worse taste perception. Smokers reported poorer subjective sweet taste perception [2.0 (IQR: 1.0–2.5) vs. 1.0 (IQR: 0–1.5); *p* = 0.027] and showed lower overall objective taste scores [9.0 (IQR: 7.0–10.0) vs. 11.0 (IQR: 7.5–12.0); *p* = 0.040].

BMI was positively correlated with worse subjective perception of salty (Rho = 0.268; *p* = 0.025) and umami tastes (Rho = 0.266; *p* = 0.026), suggesting greater impairment with increasing BMI. No significant associations were observed between taste alterations and type or duration of chemotherapy, or comorbidities.

Several associations were identified between dietary intake and taste perception. Greater impairment of bitter taste was associated with higher protein intake (Rho = 0.264; *p* = 0.028), higher alcohol consumption (Rho = 0.240; *p* = 0.045), and lower carbohydrate intake (Rho = −0.282; *p* = 0.018). Poorer perception of salty taste was correlated with higher lipid intake (Rho = 0.265; *p* = 0.027) and lower carbohydrate intake (Rho = −0.244; *p* = 0.042). Similarly, greater impairment of sour taste was associated with higher protein intake (Rho = 0.236; *p* = 0.050) and lower carbohydrate intake (Rho = −0.256; *p* = 0.033). Overall taste impairment was associated with higher lipid intake (Rho = 0.293; *p* = 0.014) and lower carbohydrate intake (Rho = −0.276; *p* = 0.021).

Objective taste measures also showed associations with dietary intake: bitter taste recognition was inversely correlated with fiber intake (Rho = −0.243; *p* = 0.043), while sweet taste recognition was inversely correlated with protein intake (Rho = −0.240; *p* = 0.046).

In multivariable regression analyses adjusted for age, education, BMI, and smoking status, carbohydrate intake was inversely associated with worsening subjective perception of overall taste (*β* = −1.96; 95% CI − 3.72 to −0.20; *p* = 0.035), as well as sour (*β* = −2.05; 95% CI −4.01 to −0.09; *p* = 0.044) and salty tastes (*β* = −1.92; 95% CI −3.76 to −0.04; *p* = 0.046).

## 4 Discussion

Women with breast cancer undergoing chemotherapy experienced taste alterations that are associated with changes in eating habits. Objective and subjective methods for assessing taste alterations showed limited agreement.

### 4.1 Taste alterations

In our patients, a statistically significant increase in taste perception difficulty was observed from baseline (prior to chemotherapy initiation) to post-treatment, as assessed by the subjective evaluation method. Taste alterations during chemotherapy have been previously described using patient-reported outcome measures (25, 26); however, differences in assessment tools and study design limit direct comparability with the present findings. In a longitudinal study including 518 patients with cancer receiving chemotherapy, taste alterations were reported by approximately 55–70% of participants using self-reported symptom questionnaires administered during treatment, although no validated taste-specific scale or baseline assessment was included (25). In a cohort of 52 women with breast cancer undergoing taxane- and anthracycline-based chemotherapy, taste alterations were reported by up to 70% of patients based on self-reported symptoms collected during treatment, without the use of validated multidimensional questionnaires or objective testing (26).

More recent prospective studies employing CiTAS have provided a more detailed characterization of taste alterations (2, 27). A cross-sectional study conducted in a mixed oncology population of 151 patients, including 74 women with breast cancer, reported taste alterations in 67.5% of participants undergoing chemotherapy; however, the absence of baseline assessments precluded evaluation of treatment-related changes over time (27). In a breast cancer–specific longitudinal cohort of 182 women, taste alterations were reported in 53% of patients during early chemotherapy cycles and increased to up to 84% during later cycles, indicating a progressive worsening with cumulative chemotherapy exposure (2). This temporal pattern closely mirrors the findings of the present study, in which subjective taste perception significantly deteriorated from baseline to post-treatment across all taste modalities.

In our study, objective taste alterations assessed by taste strips were detected in approximately one third of patients at the end of chemotherapy. Comparable prevalence rates have been reported in previous studies using the same objective method in women with breast cancer (1, 28). In a German cohort of 31 patients, overall taste alterations were identified in 37% of participants following chemotherapy (28). Similarly, a prospective cohort study conducted in Japan including 41 women with breast cancer reported objective taste alterations in 34% of patients using taste strips administered after chemotherapy cycles (1).

With respect to individual taste modalities, objective assessments in our study revealed higher rates of hypogeusia and ageusia for sour and salty tastes. A preferential impairment of sour taste perception has previously been documented using objective gustatory testing in a cohort of 44 women with breast cancer undergoing chemotherapy (29). In contrast, salty taste impairment has been more consistently reported across studies and assessment methods, being identified both through objective taste strip testing in 31 women with breast cancer (28) and through longitudinal subjective evaluation using the CiTAS questionnaire in a larger cohort of 182 women (2). In our cohort, the CiTAS questionnaire also identified salty taste as the most affected modality, suggesting partial concordance between subjective perception and objective detection for this taste quality.

Despite this alignment, the overall agreement between subjective and objective methods in our study was limited, with fair concordance observed only for sweet and salty tastes. Similar discrepancies have been reported previously. In a study evaluating awareness of dysgeusia and gustatory testing in 50 women with breast cancer, no significant agreement was observed between self-reported taste alterations and objective taste strip results across most taste modalities (18). In another prospective study including 41 women with breast cancer, concordance between subjective taste perception and objective taste testing was observed only for the salty taste modality, while no significant agreement was found for sweet, sour, or bitter tastes. This finding further supports the notion that subjective and objective approaches capture partially distinct dimensions of chemotherapy-induced taste dysfunction (1). Evidence supporting the complementary nature of subjective and objective assessments has been provided by a recent study evaluating taste alterations in 55 oncology patients, 73% of whom had breast cancer, using both objective aqueous taste solutions and the CiTAS questionnaire (19). Our findings support the use of combined assessment approaches to comprehensively characterize chemotherapy-induced taste alterations.

The partial discordance observed between subjective and objective assessments, together with the heterogeneous involvement of different taste modalities, suggests that chemotherapy-induced taste alterations arise from multiple and not yet fully elucidated mechanisms. Clinical and observational studies hypothesized that cytotoxic treatments may impair taste perception through peripheral mechanisms, including damage to the oral mucosa, salivary glands, and gustatory pathways, as well as through changes in salivary flow and composition (3, 14, 17).

Systemic inflammation and oxidative stress have also been proposed as contributing factors, potentially interfering with peripheral taste receptor function and central processing of gustatory signals (5, 17). In addition, observational studies have consistently reported an association between higher BMI and reduced taste sensitivity, and metabolic and hormonal alterations involved in appetite regulation have been proposed as possible modulators of chemosensory function, although causal relationships cannot be established (30–32).

Finally, subjective taste perception appears to be influenced by cognitive and emotional factors related to food experiences during chemotherapy, which may partly explain the limited agreement between subjective and objective assessments (19).

### 4.2 Taste alterations and individual characteristics

An inverse relationship between subjective taste perception and education level was detected in our patients. Evidence from literature is inconsistent. An analysis of data from the U.S. National Health and Nutrition Examination Survey (NHANES) 2011–2014, including 7,181 adults, found that educational level was associated with olfactory impairment but not with taste dysfunction, suggesting a differential impact of socioeconomic factors on chemosensory modalities in the general population (33). In contrast, a subsequent analysis of NHANES data from the same survey cycles, involving 5,068 adults, reported a higher prevalence of taste impairment among individuals with lower educational level and lower income levels, indicating that socioeconomic disadvantage may also be associated with reduced taste perception (34).

Smoking habits lead to a reduction in the ability to perceive tastes through a morphological alteration of the taste buds, the vascularization of the fungal papillae and through an increase in respiratory infections and dental problems (4). Consistently, our patients who were current smokers reported a lower subjective capacity in taste perception.

In our cohort, higher BMI was associated with worse subjective taste perception. A similar inverse relationship between BMI and taste sensitivity has been reported in several observational studies conducted in non-oncological populations. In a study including 206 adult participants, reduced sensitivity to multiple basic taste modalities, particularly sweet, salty, and bitter, was observed with increasing BMI, suggesting a generalized decline in taste function among individuals with overweight and obesity (32). Consistently, an observational study involving 172 adults demonstrated a progressive worsening of both taste and olfactory perception across increasing BMI categories, with higher BMI associated with altered taste perception and changes in food-related behaviours (31). Further evidence comes from a cross-sectional study of 300 adults, which reported significant differences in the perception of basic tastes across BMI classes, with individuals with higher BMI showing poorer taste perception, particularly for salty and umami tastes (30). Although causal relationships cannot be established, these associations have been interpreted in the context of metabolic and hormonal alterations accompanying increased adiposity. In particular, dysregulation of hormones involved in appetite and energy homeostasis, such as leptin, ghrelin, and insulin, has been proposed to influence both peripheral taste receptor function and central processing of chemosensory signals (30–32).

### 4.3 Taste alterations and dietary habits

In our cohort, dietary habits changed from the start to the end of chemotherapy, with a reduction in sugar and alcohol intake and a concomitant increase in protein and dietary fiber consumption, while total energy intake remained stable. A similar pattern of changes in diet composition has been reported in a prospective study including 205 women with BC undergoing chemotherapy, which documented reduced intake of sugars, alcohol, processed meats, and saturated fats, together with increased fruit consumption after treatment (35). Further analyses conducted in the same cohort showed that, although taste alterations were frequently reported, the severity of these alterations was not associated with changes in overall dietary habits or body weight over time (36). This finding suggests that patients may adopt adaptive or compensatory dietary strategies that mitigate the nutritional impact of taste dysfunction during chemotherapy (36).

Consistently, in our study, body weight and BMI did not change significantly from baseline to the end of chemotherapy, despite the observed modifications in dietary composition. However, dietary changes during chemotherapy should be interpreted cautiously, as they may reflect a combination of factors, including treatment-related symptoms, nutritional counselling, and behavioral adaptation. Indeed, energy balance during chemotherapy is influenced by factors beyond food intake, such as hormonal changes, systemic inflammation, and variations in physical activity, which may contribute to weight stability or heterogeneous weight trajectories during treatment (11, 12).

In our study, an inverse association was observed between carbohydrate intake and subjective difficulties in perceiving all tastes, as well as bitter and sour tastes. Previous studies have reported that taste alterations during chemotherapy are associated with changes in food preferences and dietary intake, including avoidance of specific foods, reduced enjoyment of meals, and modifications in macronutrient distribution, although the direction and magnitude of these changes vary considerably across studies (7, 13, 19, 37–40).

However, these findings should be interpreted with caution. Dietary intake was assessed using a food-frequency questionnaire, which may be subject to recall bias. In addition, multiple correlations were explored, increasing the risk of type I error. Furthermore, dietary changes during chemotherapy may be influenced by several factors, including nutritional counselling, treatment-related symptoms, and behavioral adaptation. Therefore, these associations should be considered exploratory and hypothesis-generating, and no causal relationship can be inferred.

### 4.4 Strengths and limitations

Our study contributes to the existing literature by evaluating taste alterations using both subjective and objective methods in a homogeneous cohort of BC patients undergoing chemotherapy. By applying a mathematical harmonization of the scales to compare these measures, we observed a low level of agreement, supporting the concept that subjective perception and objective taste function may represent distinct but complementary aspects of taste alterations. These findings highlight the importance of integrating both approaches in the clinical assessment of dysgeusia in oncology settings.

An important limitation of this study is the lack of baseline objective taste assessment. Objective assessment of dysgeusia was performed only after completion of chemotherapy (T1). The absence of clinically relevant taste alterations prior to treatment was verified at baseline through the CiTAS questionnaire. Objective taste testing was initially considered at both time points; however, during the initial phase of the study, a potential familiarization (learning) effect with the taste strips was observed, which could not be reliably distinguished from true changes in gustatory function. To minimize this potential bias and preserve the internal validity of the post-treatment assessment, objective testing was therefore performed only at T1. The factor structure of the CiTAS questionnaire was not re-evaluated in the present sample. Although the original validated subscale structure was retained, its psychometric properties were not formally assessed in this specific population, which may influence the interpretation of the results. The transformation of the CiTAS score to align with the objective scale was performed to enable direct comparison between methods; however, this approach has not been previously validated and may influence the interpretation of agreement analyses. Therefore, these results should be interpreted with caution. Finally, the analysis was limited to the ‘Taste Diminution’ subscale of the CiTAS, and other dimensions of taste alterations were not explored, which may limit the comprehensive assessment of the multidimensional nature of dysgeusia.

The relatively modest sample size represents an important limitation of this study. Although comparable to that of previous studies investigating taste alterations in cancer patients (1, 18, 19), it may have limited the statistical power of subgroup analyses and multivariable models. In addition, the number of comparisons performed may increase the risk of type I error. Therefore, these findings should be interpreted with caution and considered exploratory, and may not be fully generalizable to larger or more diverse patient populations. The threshold used to define hypogeusia (Taste Strips score <9) was derived from normative data obtained in non-oncological populations and has not been specifically validated in cancer patients, which may limit its applicability in this context. Both taste perception and dietary intake were assessed using self-reported instruments, which may be subject to recall bias and reporting inaccuracies, although validated questionnaires were employed to mitigate this limitation. Given the exploratory nature of these analyses and the multiple correlations tested, the results should be interpreted as hypothesis-generating rather than confirming a causal relationship.

## 5 Conclusion

In our study, subjective assessment showed a significant deterioration of taste perception from baseline to the end of chemotherapy, whereas objective testing identified taste impairment in approximately one third of patients, mainly affecting sour and salty tastes. The limited agreement between subjective and objective assessments supports the integration of both approaches for a more comprehensive evaluation.

Future studies are needed to determine whether patients could benefit from the use of both methods by enabling a personalized evaluation of this often-underestimated side effect, which can have a substantial impact on quality of life, and social interactions.

## Data Availability

The raw data supporting the conclusions of this article will be made available by the authors, without undue reservation.
