# Supplementary material for: Taste alterations in breast cancer patients undergoing chemotherapy: an observational study
Source: Front Nutr. 2026 Apr 22;13:1815891. doi: 10.3389/fnut.2026.1815891 (PMC13143719; doi:10.3389/fnut.2026.1815891)
Supplement: Supplementary file 1 [file Table_1.DOCX]

**Supplementary material**

**Figure 1s.** Sequence of presentation of the taste strips to the user.


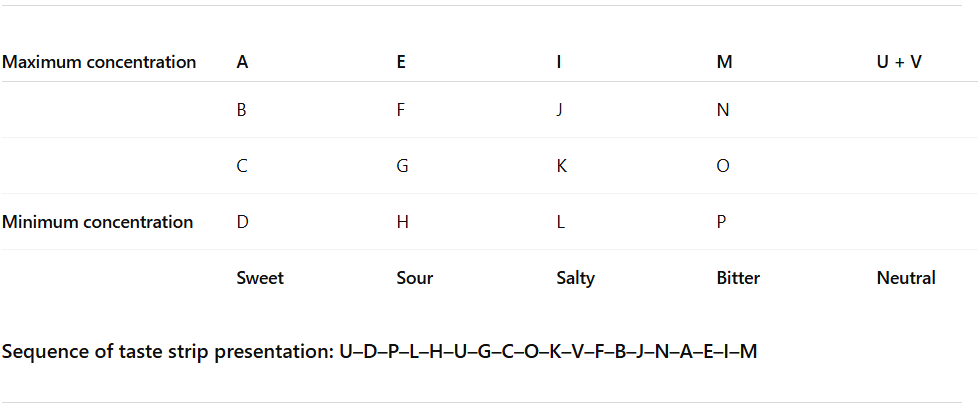


**Figure 2s.** Flow of the study

**
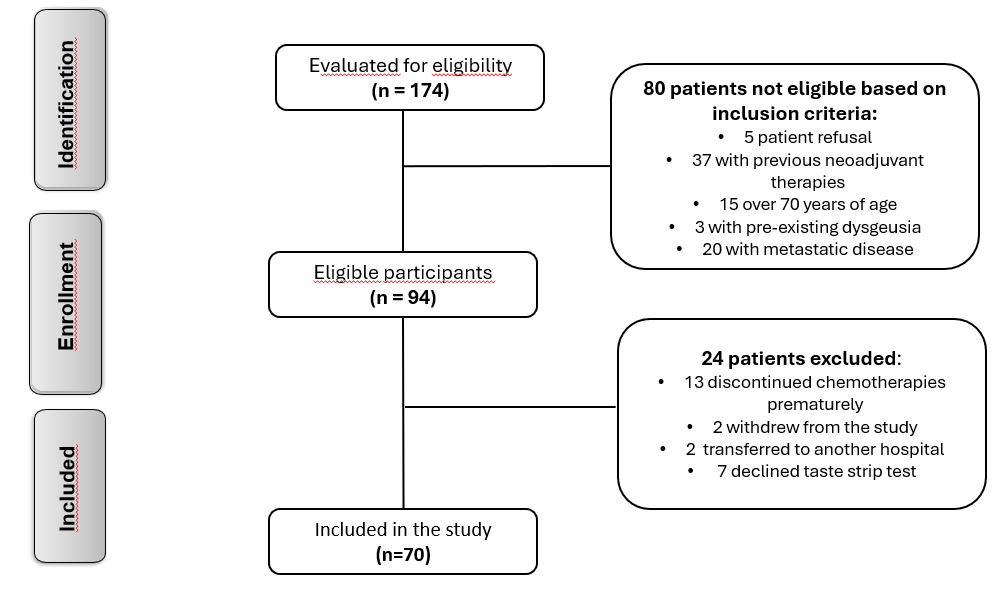
**

**Table 1s.** Changes in taste perception (ΔT1–T0) (subjective method) according to level of education.

| **Δ (T1 -T0)**  **Taste alterations (CITAs)** | **Primary schools** | **P within group** | **High school** | **P within group** | **Bachelor/**  **Master’s degree** | **P within group** | **P**  **between**  **groups** |
| --- | --- | --- | --- | --- | --- | --- | --- |
| Sweet | + 1.0 (1-2) | **<0.0001** | + 1 (0-2.0) | **<0,0001** | 0 (0-1.25) | **<0,0001** | **0.043** |
| Sour | + 1.5 (1-2) | **<0.0001** | + 1.0 (0.75-2.0) | **<0,0001** | 0 (0-1.0) | **0.001** | **0.003** |
| Bitter | + 1.0 (1-2) | **0.001** | + 1.0 (0 -2.0) | **<0,0001** | 0 (0-1.0) | **0.002** | **0.004** |
| Umami | + 2.0 (1-2.75) | **<0.0001** | + 1.0 (1.0-3.0) | **<0,0001** | + 1.0 (0-2.0) | **<0,0001** | **0.042** |

**Note:** Data are presented as median (Q1–Q3). Statistical analysis was performed using Wilcoxon signed-rank test (p values within group) and the Kruskal–Wallis test (p values between groups).
